# Immunisation of Sheep with Bovine Viral Diarrhoea Virus, E2 Protein Using a Freeze-Dried Hollow Silica Mesoporous Nanoparticle Formulation

**DOI:** 10.1371/journal.pone.0141870

**Published:** 2015-11-04

**Authors:** Donna Mahony, Karishma T. Mody, Antonino S. Cavallaro, Qiuhong Hu, Timothy J. Mahony, Shizhang Qiao, Neena Mitter

**Affiliations:** 1 Queensland Alliance for Agriculture and Food Innovation, The University of Queensland, St Lucia, QLD, 4072, Australia; 2 School of Chemical Engineering, The University of Adelaide, SA, 5005, Australia; The Ohio State University, UNITED STATES

## Abstract

Bovine viral diarrhoea virus 1 (BVDV-1) is arguably the most important viral disease of cattle. It is associated with reproductive, respiratory and chronic diseases in cattle across the world. In this study we have investigated the capacity of the major immunological determinant of BVDV-1, the E2 protein combined with hollow type mesoporous silica nanoparticles with surface amino functionalisation (HMSA), to stimulate immune responses in sheep. The current work also investigated the immunogenicity of the E2 nanoformulation before and after freeze-drying processes. The optimal excipient formulation for freeze-drying of the E2 nanoformulation was determined to be 5% trehalose and 1% glycine. This excipient formulation preserved both the E2 protein integrity and HMSA particle structure. Sheep were immunised three times at three week intervals by subcutaneous injection with 500 μg E2 adsorbed to 6.2 mg HMSA as either a non-freeze-dried or freeze-dried nanoformulation. The capacity of both nanovaccine formulations to generate humoral (antibody) and cell-mediated responses in sheep were compared to the responses in sheep immunisation with Opti-E2 (500 μg) together with the conventional adjuvant Quil-A (1 mg), a saponin from the Molina tree *(Quillaja saponira*). The level of the antibody responses detected to both the non-freeze-dried and freeze-dried Opti-E2/HMSA nanoformulations were similar to those obtained for Opti-E2 plus Quil-A, demonstrating the E2 nanoformulations were immunogenic in a large animal, and freeze-drying did not affect the immunogenicity of the E2 antigen. Importantly, it was demonstrated that the long term cell-mediated immune responses were detectable up to four months after immunisation. The cell-mediated immune responses were consistently high in all sheep immunised with the freeze-dried Opti-E2/HMSA nanovaccine formulation (>2,290 SFU/million cells) compared to the non-freeze-dried nanovaccine formulation (213–500 SFU/million cells). This study is the first to demonstrate that a freeze-dried silica mesoporous nanovaccine formulation gives balanced immune responses in a production animal.

## Introduction

Bovine viral diarrhoea virus 1 (BVDV-1) infection occurs in the target species of cattle and sheep herds worldwide and therefore remains of economic importance. BVDVs are a group of positive sense, single-stranded RNA viruses classified in the *Pestivirus* genus within the Flaviviradae family [1]. The BVDV-1 genome is transcribed as a single, large (~12.3 kb) open reading frame which is translated into a single polyprotein, and processed into individual viral proteins by viral and cellular proteases [2].

Currently there is no commercially available recombinant subunit vaccine for BVDV-1, only modified live or inactivated vaccines. The E2 membrane glycoprotein has been shown to be the major immunogenic protein of BVDV-1 [2] and is the viral antigen that is efficiently recognised by the immune system [3]. Therefore E2 has been the focus as a potential candidate for the development of a subunit BVDV-1 vaccine in a number of studies [4–8].

Subunit vaccines often require the addition of an adjuvant which potentiates the immune response to the protein antigen in the vaccinated host. The role of mesoporous silica nanoparticles (MSNs) as an antigen carrier and adjuvant has been recently been reviewed [9, 10]. MSNs are proving to be a valuable alternative to conventional adjuvants such as aluminium hydroxide (or alum) which can have adverse effects at the injection site when administered subcutaneously or intra-dermally [11]. Various types of silica nanoparticles have been used to deliver antigens in immunisation studies that have induced both humoral and cell-mediated immune responses [12–16]. Injection of MSNs showed no local reactions at the injection site both at a gross and histopathological level and they are well tolerated in the mammalian system [16–18].

Recently we have demonstrated that E2 delivered by amino functionalised hollow mesoporous silica nanoparticles generated balanced immune responses in mice with both antibody and cell-mediated immunity [17]. Here, we expand on our previous work by developing a freeze-dried process for E2 adsorbed hollow type MSN with surface amino functionalisation (HMSA) and compare the immunogenicity of the non-freeze dried and freeze-dried nanoformulations in sheep. To the best of our knowledge this is the first time that silica based nanoparticles have been used in a large animal model. Immunisation of sheep with the E2 nanovaccine did not show any adverse effects on animal health and produced both humoral and cell-mediated immune responses. Importantly, the long term cell-mediated immune responses were detectable up to four months after immunisation and were higher in the sheep immunised with the freeze-dried E2 nanovaccine formulation.

## Material and Methods

### Preparation of amino functionalised hollow mesoporous silica nanoparticles (HMSA)

The nanoparticles used in this study were hollow mesoporous silica nanoparticles with amino groups added to the particle surface. The synthesis method of the HMSA nanoparticles used in this study has been described previously [17]. To obtain a monodisperse suspension, nanoparticles (100 mg) were dispersed in 50 mM Tris (pH 7.0, 0.2% Igepal CA630 (10 mL) and ultrasonicated in a glass vial for 1 min at ambient temperature (25°C) using a 5mm probe sonicator (Hielscher UP100H, Teltow, Germany) at 60% amplitude.

### Adsorption of Opti-E2 to HMSA for freeze-drying

An *Escherchia coli* codon-optimised, truncated version of E2 (which lacks the membrane binding domain of native BVDV E2) was expressed, purified, endotoxin-treated and then solubilised as described previously for Opti-E2 protein [19].

Suspensions of HMSA (10 mg/mL in 50 mM Tris (pH7.0), 0.2% Igepal CA630, (Sigma Aldrich, St. Louis, USA) were prepared as described above. Adsorption of Opti-E2 protein on HMSA were performed at 25°C using 200 μg of Opti-E2 and 2 mg HMSA (10 mg/ mL) in 50 mM Tris, 0.2% Igepal CA630 buffer (pH7.0) at 200 rpm for 22 hr. A 200 μL sample of the particle-protein slurry was removed and centrifuged (16.2 g, 1 min). The amount of Opti-E2 protein remaining in the adsorption supernatants were visualised by gel electrophoresis and quantified using a microtitre plate format protein assay kit (Biorad DC kit, Hercules, USA) following the manufacturer’s instructions.

### Freeze-drying process

Following Opti-E2 adsorption to the HMSA particles, the samples were centrifuged at 4500 *g* for 5 min and the supernatants were removed. Different combinations and concentrations of excipients were added to the Opti-E2/HMSA pellets prior to freeze-drying and the final volume adjusted to 1 mL. Samples were frozen in liquid nitrogen then placed in a freeze-dryer (Martin Christ Model LPC-32, Osterode AM Harz, Germany) at 24°C, 0.11 mbar for 20 hr for the primary drying step. The secondary drying step was at 24°C, 0.01 mbar for 2 hr. Freeze-dried samples were stored in a vacuum desiccator at ambient temperature.

### Reconstitution and transmission electron microscope (TEM) of lyophilised samples

Samples were reconstituted in 1 mL sterile PBS. The physical characteristics of the freeze-dried nanoparticles were observed using transmission electron microscopy (TEM) before and after freeze-drying.

### SDS-PAGE Electrophoresis

SDS-PAGE analysis was performed using XCell *SureLock* ^®^ Mini-Cell precast system (Invitrogen, Carlsbad, USA) with NuPAGE 10% BIS-Tris gels according to manufacturer instructions. Size estimations were determined against SeeBlue^®^ Plus2 (Invitrogen) pre-stained molecular weight standards. The resolved proteins were visualised by staining in 50% methanol, 10% acetic acid, 0.25% Coomassie Blue R250 for 30 min, followed by destaining in 30% methanol, 10% acetic acid for 10 min three times.

### Screening of sheep to determine BVDV status

Forty sheep were purchased from using a commercial agent from a property at Mungindi on the border between Queensland and New South Wales, Australia. All the procedures involving the animals were approved by The Department of Agriculture, Fisheries and Forestry Animal Ethics Committee. Ethics approval number SA 2011/05/358. The sheep were housed in 3 x 3 m pens with 4 animals per pen (in accordance with the Animal Ethics guidelines) for the first 3 months of the study and then the sheep were put out to pasture for the remaining 4 months of the study.

Since BVDV-1 is endemic in many sheep herds it was essential to screen the animals to determine whether they were currently infected or had been previously infected with BVDV-1 to determine their suitability for inclusion in the study.

The sheep (n = 40) were screened for BVDV-1 infection status using quantitative real time PCR as described previously [20]. The sheep were also screened for the presence of BVDV IgG antibodies using an E2-specific ELISA (described below). Sera samples from the sheep were diluted from 1:100 to 1:6400 for the ELISA assays. The ELISA results showed that 23 out of the 40 had an absorbance reading of less than 0.1 at 690 nm (data not shown) at 10^2^ dilution of the sera samples. This showed there was a no response to E2 and therefore these animals were designated BVDV negative. The other 17 animals had a range of absorbance readings from >0.1 to 0.4 at 10^2^ dilution of the sera samples. For the immunisation trial 16 animals with no E2 ELISA response were randomly assigned into 4 groups (4 sheep per group).

The sheep were closely monitored throughout the study and weighed routinely to monitor changes in health. All the animals remained in good health for the duration of the 7 month study with no visible deleterious health effects. The average weight range of the sheep at start of trial was 37 kg and at the end of the trial was 49 kg.

### Immunisation of sheep

Four animals were allocated into each of the four treatment groups (Table 1). Opti-E2 protein adsorption to HMSA was performed within 24 hr of animal immunisation.

**Table 1:** The immunisation groups in the sheep study.

Treatment Group	Group Description	Injected dose (1 mL)
1	Opti-E2	500 μg Opti-E2 + 1 mg Quil-A
2	Opti-E2/HMSA	500 μg Opti-E2/6.2 mg HMSA
3	Freeze-dried Opti-E2/HMSA	500 μg Opti-E2/6.2 mg HMSA
4	HMSA only	6.2 mg HMSA

Injection doses of the E2 nanovaccine were prepared by isotherm adsorption reactions.

The Opti-E2/HMSA adsorption reactions were prepared aseptically using 500 μg Opti-E2 protein and 6.2 mg HMSA (10 mg/mL) in sterile 50 mM Tris, 0.2% Igepal CA630 buffer (pH 7.0) in a total volume of 5 mL at 25°C, 200 rpm for 22 hr. Following adsorption the particles were centrifuged at 4500 *g* for 5 mins and the supernatant was removed.

Freeze-dried Opti-E2/HMSA nanoformulation included 5% trehalose and 1% glycine. Samples were frozen in liquid nitrogen then freeze-dried as described above. Freeze-dried samples were stored in a vacuum desiccator at ambient temperature (25°C) before use.

Quil-A (2 mg/mL, Superfos Biosector, Vedback, Denmark) was resuspended in sterile injectable water (Pfizer). The doses were resuspended in 1 mL injectable saline (0.9%) and administered by subcutaneous injection at the base of the ear using a sterile 23 gauge needle. Three injections were administered at three week intervals. The injection doses (Table 1) administered were Opti-E2 plus Quil-A (500 μg Opti-E2 and 1 mg Quil-A), non-freeze-dried Opti-E2-HMSA (500 μg/6.2 mg HMSA), freeze-dried Opti-E2-HMSA (500 μg/6.2 mg HMSA) and HMSA (6.2 mg).

Pre-immune (PI) blood samples were collected prior to immunisation and subsequent samples were collected at two-week intervals following each injection and then at monthly intervals for 4 months after the final injection. Blood samples were collected via jugular vein collection using 20 gauge needle into lithium-heparin vacutainer tubes (Becton Dickinson, New Jersey, USA).

### E2-specific ELISA

ELISAs for the detection of Opti-E2-specific antibodies were performed by coating microtitre plates (Nunc Maxisorb, Roskilde, Denmark) with 50 μL Opti-E2 antigen solution (2 ng/μL) in PBS overnight at ambient temperature. The coating solution was removed and the plates were washed once with PBS-T (1x PBS, 0.1% Tween-20, Sigma Aldrich) and then blocked with 1% Bovine Serum Albumin (Sigma Aldrich) and 1% skim milk (Fonterra, Auckland, New Zealand) in 200 μL PBS for 1 hr with gentle shaking at ambient temperature. Plates were washed three times with PBS-T.

Sheep sera samples were diluted from 1:100 to 1:6400 in 50 μL PBS and added to the wells of the blocked plates followed by incubation for 2 hr at ambient temperature. To detect sheep antibodies 100 μL HRP conjugated monoclonal anti-sheep IgG antibodies (Sigma Aldrich) diluted in PBS to 1:40,000 were added to each well and incubated for 1 hr at ambient temperature with gentle shaking. The plates were washed three times in PBS-T and 100 μL TMB substrate (Life Technologies) was added and incubated for 15 min; 100 μL of 1 N HCl was added to the wells to stop the chromogenic reaction. The plates were read at 450 nm on a BioTek microplate reader (Winooski, US).

### Isolation of peripheral blood mononucleocytes and interferon-γ (IFN-γ) ELISPOT assay

To 35 mL whole blood in a 50 mL tube (Falcon), 15 mL of D-PBS (Invitrogen) was added. The samples were mixed gently by inverting the tubes. The diluted blood was gently added on top of 15 mL of Ficoll-Paque PLUS (GE Healthcare, Buckinghamshire, United Kingdom). Samples were centrifuged at 1000 *g* for 40 min at ambient temperature in a benchtop swing-out centrifuge (Sigma, settings were “No brake” and medium acceleration). Following centrifugation, the upper plasma layer was drawn off, leaving the lymphocyte layer undistributed at the interface. The lymphocyte layer was transferred and D-PBS up to 50 mL was added and centrifuged at 450 *g* for 10 min at ambient temperature (maximum brake and acceleration). The supernatant was removed leaving 1 mL volume. Red blood cells were lysed by the addition of 9 mL of 0.17 M ammonium chloride pH 7.5 and the cell pellets were resuspended by gentle shaking. The samples were incubated at 4°C for 10 min, then 30 mL of D-PBS was added, followed by centrifugation at 800 *g* (maximum brake and acceleration) for 10 min at ambient temperature. The cells were resuspended in complete RPMI-40 media and the viable cell number was determined by trypan blue (0.2%) staining. IFN-γ ELISPOT assays was performed using the kit, ELISPOT^PLUS^ for Bovine/Ovine/Equine IFN-γ (MabTech, Stockholm, Sweden) according to manufacturer’s specifications. PBMC cells from each sheep were seeded in triplicate into Polyvinylidene fluoride (PVDF) ELISPOT plates coated with monoclonal bovine interferon-γ (IFN-γ) capture antibody. Cells were incubated in complete RPMI-40 medium at 37°C and 5% CO_2_ for 40 hr in the presence or absence of Opti-E2 protein (10 μg/mL dialysed in PBS) or the polyclonal activator concavalin A (1 μg/mL, Sigma Aldrich) as a positive control. The ELISPOT plates were read on an ELISPOT reader (Autoimmun Diagnostika, Strassburg, Germany).

### Statistical analyses

Statistical analysis of the ELISA data was performed on the average OD values (at 450 nm) of individual animals in each group (serum dilution of 1:200). The ELISA results were analysed by one-way analysis of variance and significant differences between groups were determined using Tukey’s HSD test (GraphPad Prism for Windows V5.04).

Statistical analysis of the ELISPOT data was performed on the number of SFU/million cells obtained for individual animals using an unpaired, two-tailed Student’s t-test (Microsoft Excel).

## Results and Discussion

### Characterisation of HMSA

**Fig 1:**
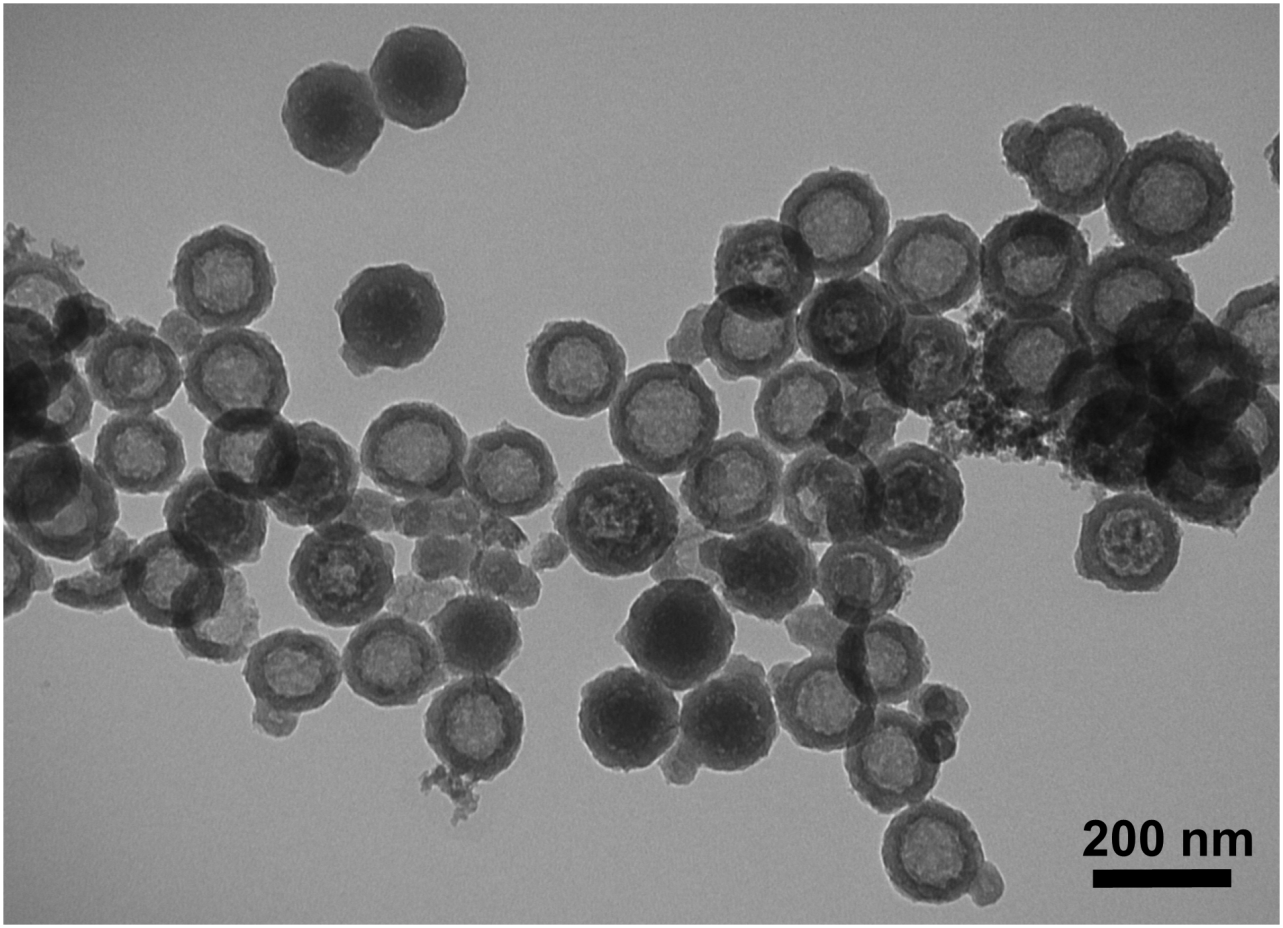
The morphology of HMSA observed by transmission electron microscopy.

**Fig 2:**
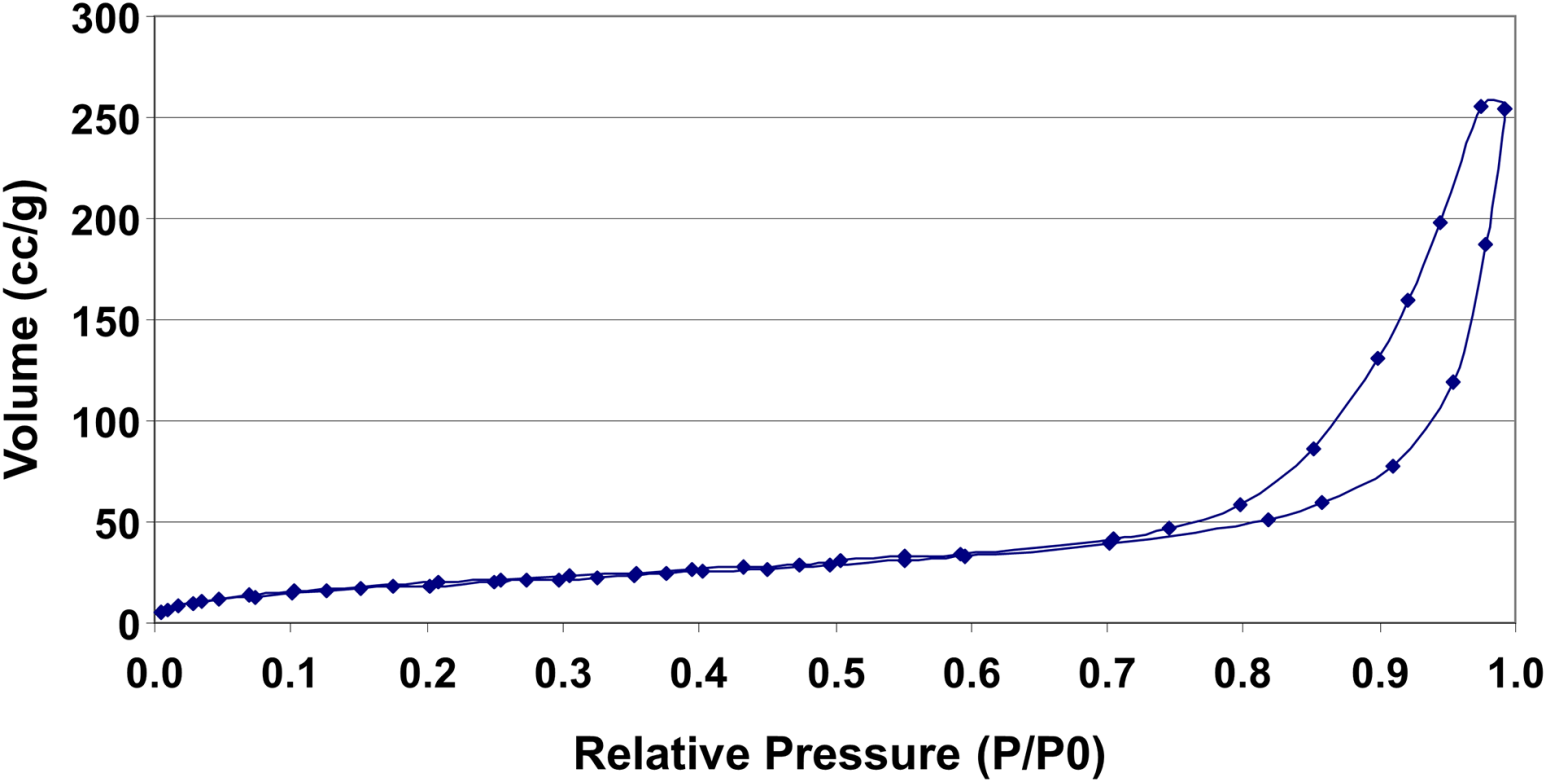
N_2_ adsorption-desorption isotherms of HMSA.

The HMSA were synthesised as described in our previous study with an E2 nanovaccine in mice [17]. The particles were of a uniform shape and size of 140 to 150 nm (Fig 1) as determined by size distribution analysis of transmission electron microscopy (TEM) images (S1 Fig). These particles were larger than the HMSA used in the previous study (120 nm) although the thickness of the particle shell was the same at 20 nm. The pore structure of HMSA was characterised by nitrogen (N_2_) adsorption-desorption isotherms (Fig 2). The nanoparticle surface area which was calculated by the Barret-Emmett-Teller (BET) method was 71.04 m^2^ g^-1^ and the total pore volume was 0.39 m^3^ g^-1^. The elemental analysis showed atomic percentages for nitrogen, carbon and hydrogen were 0.93%, 24.12% and 4.91% respectively.

### Freeze Drying of Opti-E2 adsorbed HMSA

The optimal combination of cryoprotectant and lyoprotectant that will preserve both the protein and the nanoparticles during the freeze-drying process needs to be empirically determined for each protein and nanoparticle combination. The Opti-E2 protein was bound to HMSA using isotherm conditions which gives 80 μg Opti-E2/mg HMSA [17]. Following adsorption the excipients were added to the Opti-E2/HMSA and the nanoformulations were rapidly frozen in liquid nitrogen and subsequently freeze-dried.

**Fig 3:**
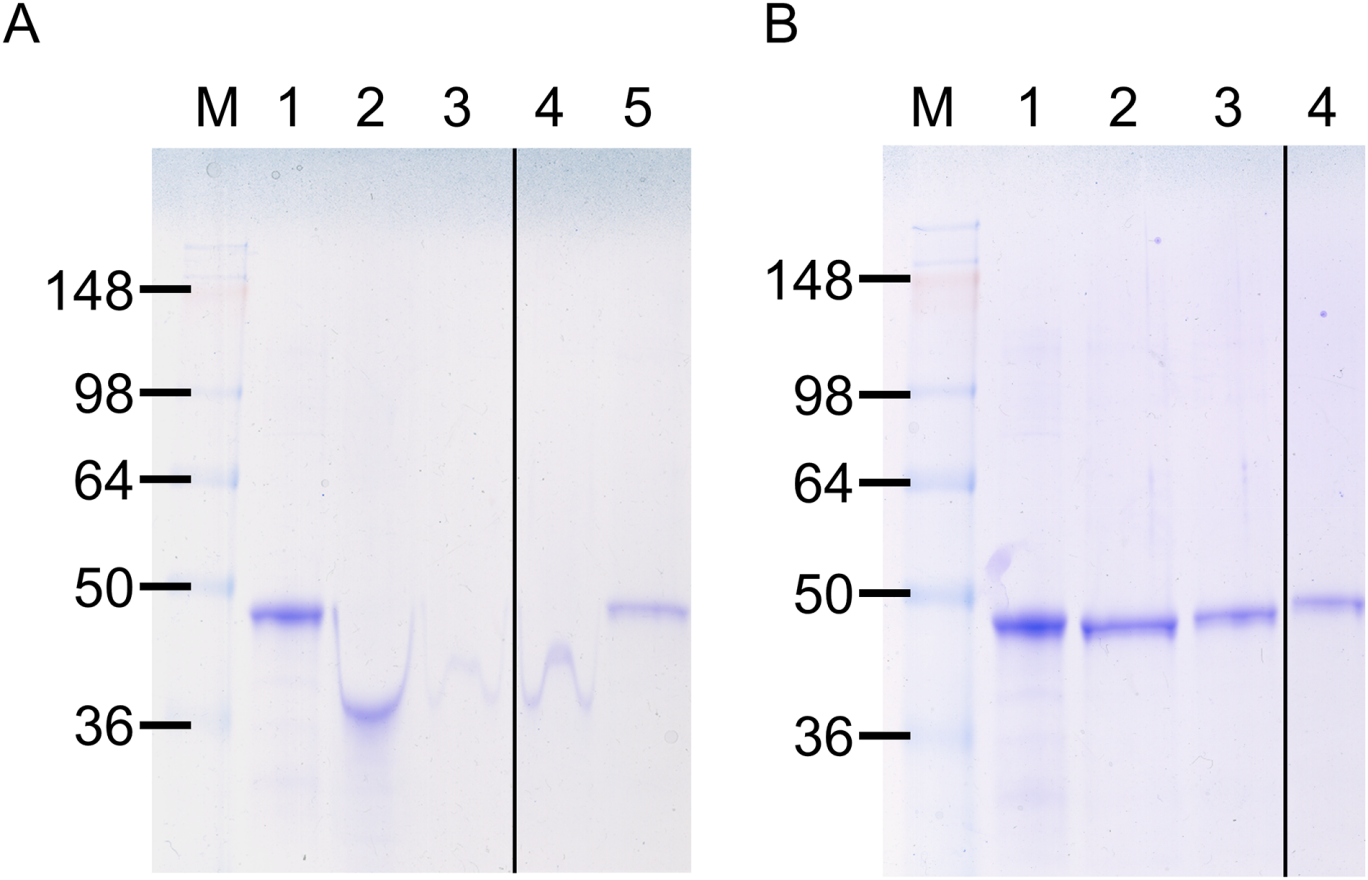
**(A)**: Evaluation by SDS-PAGE of Opti-E2/HMSA formulations after freeze-drying with different combinations of trehalose and PEG8000. Lane 1: Opti-E2 control (4 μg); Opti-E2/HMSA freeze-dried with Lane 2: 1% PEG8000; lane 3: 5% trehalose and 0.5% PEG8000; lane 4: 5% trehalose and 0.1% PEG8000. **(B)**: Evaluation by SDS-PAGE of Opti-E2/HMSA formulations after freeze-drying with different combinations of trehalose and glycine. Lane 1: Opti-E2 control (4 μg); Opti-E2/HMSA freeze-dried with lane 2: 5% trehalose and 1% glycine; lane 3: 5% trehalose and 0.5% glycine; lane 4: 5% trehalose and 0.1% glycine.

Previously we have shown successful freeze-drying of ovalbumin adsorbed mesoporous silica nanoparticles using 5% trehalose and 1% PEG8000 [21]. Therefore the first combination that was trialled with Opti-E2 bound HMSA was 5% trehalose with either 1%, 0.5% or 0.1% PEG8000. The integrity of the protein after freeze-drying was assessed by SDS-PAGE analysis of the reconstituted samples. The presence of PEG8000 in the samples distorted the electrophoretic migration of protein in SDS-PAGE gels, causing the protein to migrate at an apparently lower molecular weight. In the absence of PEG, the Opti-E2 molecular weight was approximately 42 kDa (Fig 3A, Lane 1), while in the presence of PEG8000 the molecular weight was estimated to be 30 kDa (Fig 3A, Lane 2). After freeze-drying with the excipients, 5% trehalose and 1% or 0.5% PEG8000, the migration of Opti-E2 was distorted and the protein appeared degraded as the intensity of the band was lower (Fig 3A, Lanes 3 and 4) compared to the control Opti-E2 protein (Fig 3A, Lane 1). The Opti-E2/HMSA sample freeze-dried with 5% trehalose and 0.1% PEG8000 showed Opti-E2 protein of 42 kDa (Fig 3A, Lane 5). This lower concentration of PEG8000 did not affect the electrophoretic migration of Opti-E2. However as with the higher concentrations of PEG8000 the protein was also degraded, as suggested by the lower intensity of the band compared to the control Opti-E2 protein band (Fig 3A). These results suggested that trehalose and PEG8000 were not able to stabilise the Opti-E2 during either the freeze-drying or reconstitution processes and therefore a different lyoprotectant was required.

**Fig 4:**
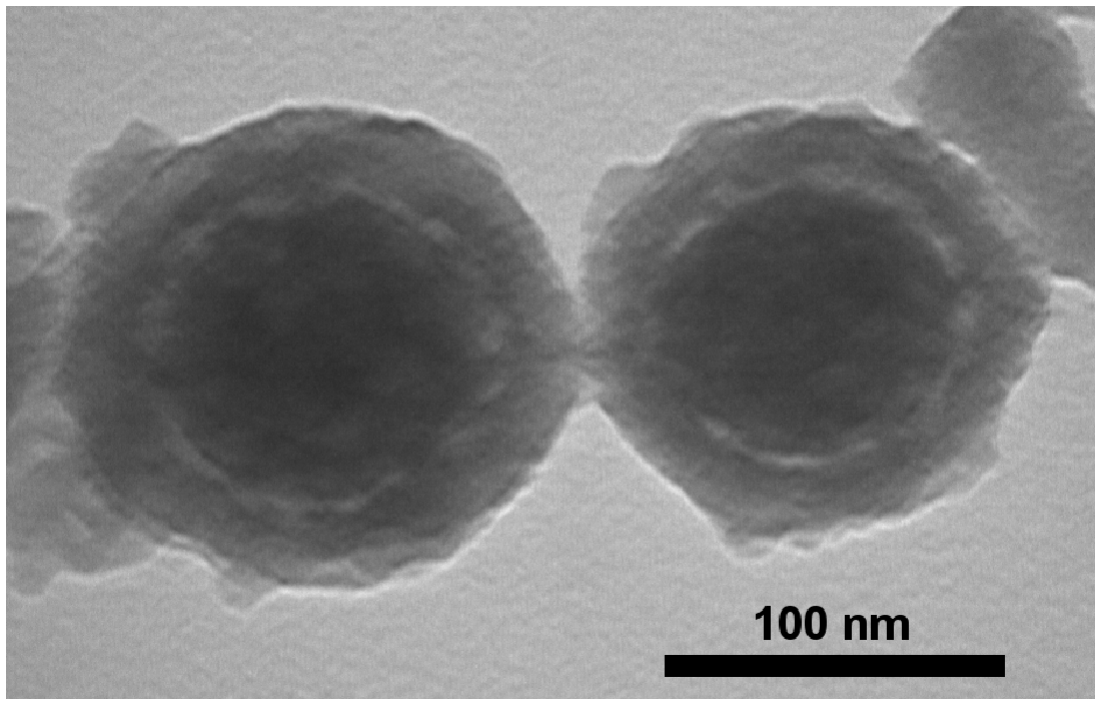
The morphology of Opti-E2/HMSA particles visualised by transmission electron microscopy following freeze-drying with 5% trehalose and 1% glycine.

Glycine was then tested as an alternative lyoprotectant since it has been used successfully for freeze-drying of other proteins such as lactate dehydrogenase and glucose 6-phosphate dehydrogenase in a sucrose-glycine based excipient system [22]. Glycine was used at 1%, 0.5% and 0.1% in combination with 5% trehalose in the freeze-drying formulations. Reconstituted, freeze-dried Opti-E2/HMSA samples were analysed on SDS-PAGE gels and showed that the Opti-E2 protein integrity was maintained at all three concentrations of glycine used (Fig 3B). Therefore 1% glycine was selected for freeze-drying of the nanovaccine formulation. The integrity of the nanoparticles following the freeze-drying process with 5% trehalose and 1% glycine was confirmed by TEM analyses. The resulting images showed that the HMSA remain intact and maintained their characteristic round shape and original size of 140–150 nm (Fig 4). The freeze-dried nanovaccine reconstituted easily and rapidly in less than 30 seconds. Furthermore, it was found that this excipient combination of 5% trehalose and 1% glycine for freeze-drying of Opti-E2/HMSA maintained the protein integrity after long term storage (14 months) at ambient temperature, whereas freeze-drying with 5% trehalose and 1% PEG8000 resulted in completely degraded protein (S2 Fig).

### Immunisation of sheep with Opti-E2 nanovaccine formulations

To determine if HMSA can act as a useful delivery vehicle for Opti-E2 protein, in livestock animals, sheep were immunised three times at three week intervals. Freeze-drying is a relatively harsh process but can be used to generate nanoparticle preparations which have long term stability at ambient temperatures [23, 24]. To determine if freeze-drying affected the activity of the Opti-E2 nanoparticle formulation, it was tested in animals both before and after freeze-drying. The sheep trial comprised of 16 BVDV-1 negative animals divided into four groups of 4 animals each, as described in Table 1.

Pre-immune (PI) sera samples were collected prior to immunisation and subsequent sera samples were collected two weeks after each immunisation. Opti-E2 antigen alone was not included as a group since protein/antigen was considered highly unlikely to produce an immune response when injected in the absence of an adjuvant [25]. We included the control group, HMSA only, as adjuvant alone is most commonly use control in large animal studies and furthermore determining the adjuvanting capacity of HMSA was the principle aim of our study [6].

**Fig 5:**
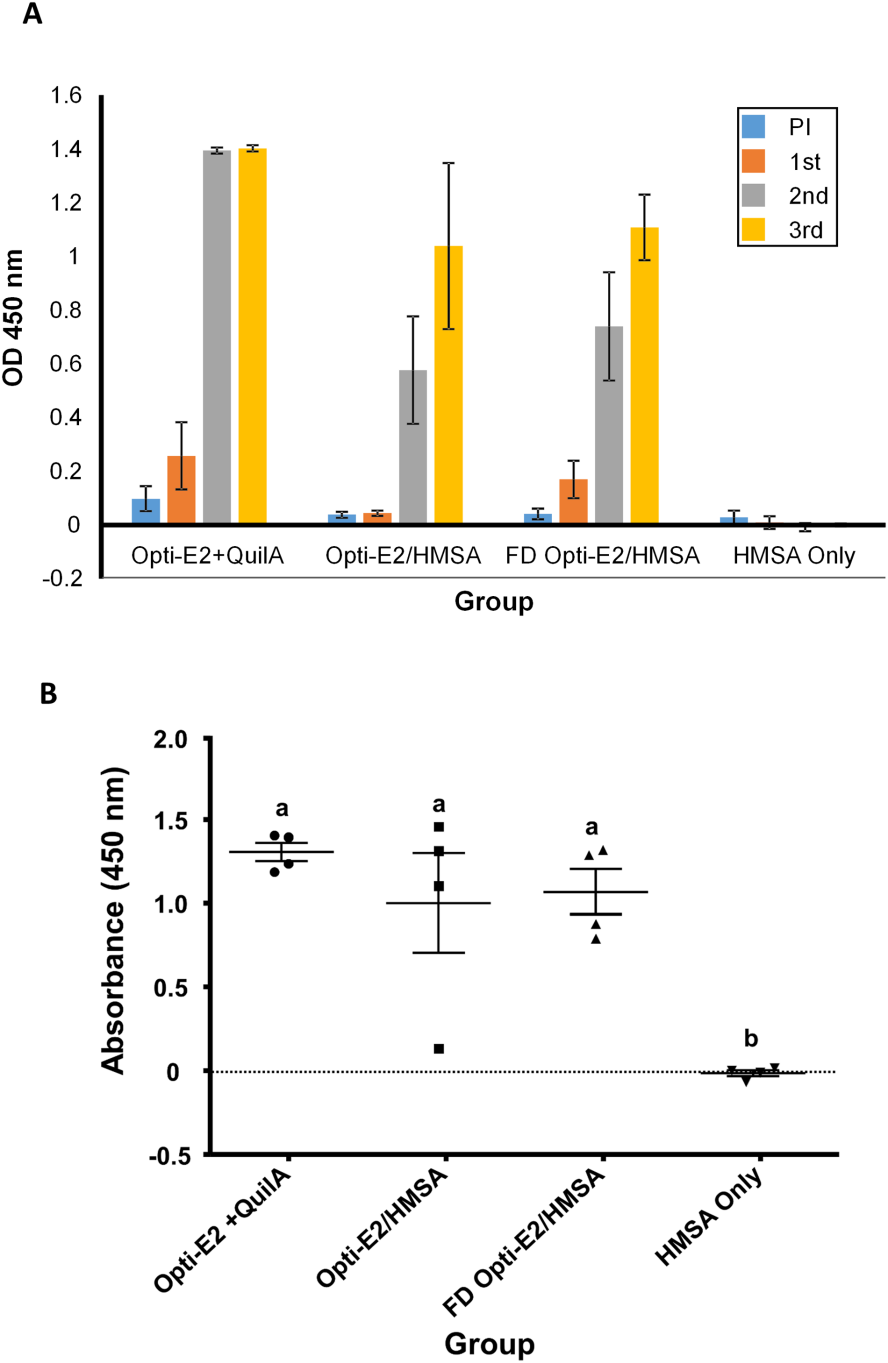
Group 1 received 500 μg Opti-E2 and 1 mg Quil-A; Group 2 received the non-freeze-dried E2 nanovaccine (500 μg Opti-E2 adsorbed to 6.2 mg HMSA), Group 3 received the freeze-dried (FD) E2 nanovaccine (500 μg Opti-E2 adsorbed to 6.2 mg HMSA), Group 4 received HMSA particles (6.2 mg) only. **(A)** Average Opti-E2-specific ELISA antibody responses (n = 4) in sheep in pre-immune sera (PI), and after one (1st), two (2nd) and three (3rd) subcutaneous immunisations. **(B)** Opti-E2-specific ELISA antibody responses in sheep after three subcutaneous immunisations. The individual response for each sheep is shown two weeks after the third immunisation using a sera dilution of 1:200.

Immune responses measured by an E2-specific Enzyme-linked Immunosorbent Assay (ELISA) showed E2-specific antibodies were detectable following two injections in all groups (Fig 5A). The total IgG responses for Opti-E2/HMSA and FD Opti-E2/HMSA were highest two weeks after the third injection (Fig 5A). No antibody response was observed for the animals injected with HMSA alone.

Further analysis of the sera at 1:200 dilution after 3 injections showed that animals immunised with Opti-E2 plus Quil-A recorded similar antibody responses (average OD value of 1.4) in all four sheep, indicating Opti-E2 protein was immunogenic in a large animal model.

For sheep receiving the Opti-E2/HMSA nanoformulation, 3 out of the 4 animals show OD values of 1.1 to 1.4 (Fig 5B). One of the animals in this group showed a low antibody response as a result of not receiving the full booster dose during the second immunisation. All four animals immunised with the FD Opti-E2/HMSA nanoformulation also showed strong antibody responses (OD values from 0.83 to 1.33). This result is important since it has demonstrated that freeze-drying of the Opti-E2 nanoformulation with the excipients 5% trehalose and 1% glycine maintained the immunological integrity of E2 protein. The control group immunised with HMSA particles showed no specific antibody response to Opti-E2 protein (Fig 5B). These results confirmed that nanoformulations can deliver E2 antigen and act as a self-adjuvant in large animals without the need of a conventional adjuvant. There was no significant difference in the antibody response of the groups receiving Opti-E2 plus Quil-A, Opti-E2/HMSA and FD Opti-E2/HMSA (Fig 5B), indicating HMSA is acting as a self-adjuvant.

The injections of Opti-E2/HMSA in the sheep at the base of the ear did not result in localised skin redness indicating that the subcutaneous route of immunisation of HMSA nanoparticles was well tolerated in sheep. The sheep remained healthy and within the normal weight range throughout the experimental period (data not shown). The route of immunisation together with adjuvant and the type of antigen itself are important considerations as they can influence the type of immunity generated in response to the vaccine antigen. The subcutaneous route of immunisation was selected based on our previous studies in mice with Opti-E2 adsorbed to HMSA [17]. It has been demonstrated that immunisation of sheep with 50 nm polystyrene beads was most effective at inducing both cellular and humoral immunity when administered through intradermal and subcutaneous routes [26]. The effectiveness of the Opti-E2 nanovaccine formulation using this route of immunisation may result from slower antigen desorption from the subcutaneous injection site creating a depot effect and therefore prolonging the immune response. The BVDV-1 vaccine, Pestigard^®^ used in Australia, is also administered subcutaneously in cattle, therefore subcutaneous delivery of nanovaccine formulations should be a viable immunisation strategy for industry use.

### Long term cell-mediated immune responses to Opti-E2/HMSA immunisation by ELISPOT assay

**Fig 6:**
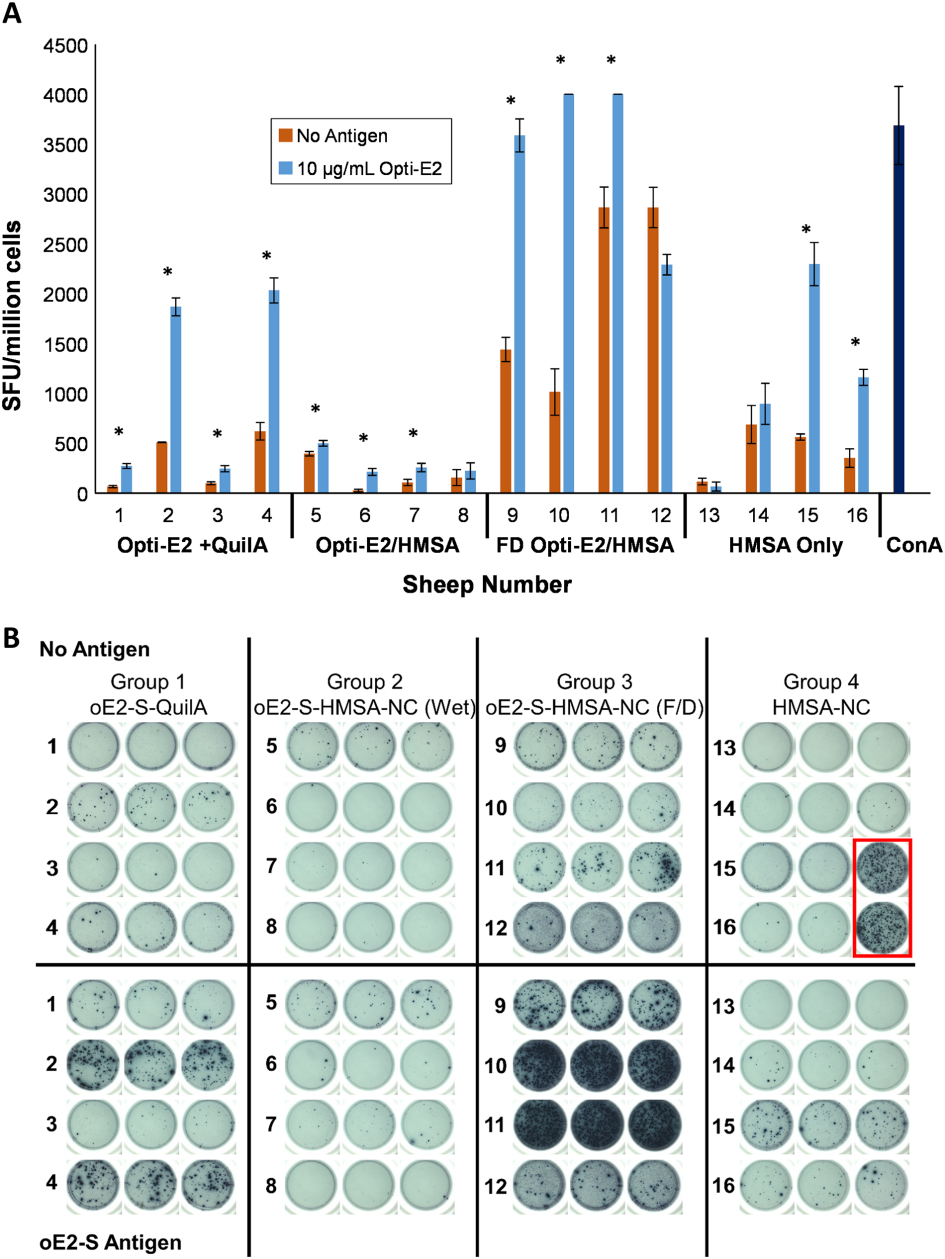
**(A)** The long term immune memory response of sheep PBMC cells following stimulation to Opti-E2 antigen. IFN-γ secretion of PBMC cells obtained four months after immunisation was assessed by ELISPOT assay in response to Opti-E2 (10 ng/μL, blue bars) and compared to unstimulated cells (red bars). The Mean Spot SFU /million cells is shown for each animal (assayed in triplicate) in the treatment groups. The polyclonal activator, Concavalin A (dark blue bars, ConA), was used to confirm cell viability and functionality of the assay. The asterisk (*) indicates significant responses with *p <* 0.05 (unpaired t-test analysis) compared to the unstimulated controls. **(B)** ELISPOT plate of the long term immune memory response of sheep PBMC cells following stimulation to Opti-E2 antigen compared to unstimulated cells. The polyclonal activator, Concavalin A (Red Box), was used to confirm cell viability and functionality of the assay.

The cell-mediated memory response to an antigen is a vital component of the immune system since it demonstrates uptake of the antigen by professional antigen presenting cells and presentation within the lymphoid organs, an essential process for developing immunity to invading pathogens. To determine if there were long term cell-mediated immune responses in sheep after immunisation using HMSA, the peripheral blood mononucleocyte cell (PBMC) populations were isolated four months following the third immunisation. Stimulated PBMCs were then used in IFN-γ Enzyme-linked Immunosorbent Spot (ELISPOT) assay to determine if there was T-helper type 1 (Th1) cell-mediated immune response. Fig 6 shows the IFN-γ response of individual sheep as indicated by the number of cells producing Spot Forming Units (SFU).

The four animals receiving Opti-E2 plus Quil-A showed some variation in the responses. Sheep 1 and 3 showed lower level responses of 244 and 272 SFU/million cells and Sheep 2 and 4 showed high level responses of 1869 and 2032 SFU/million cells in response to Opti-E2 antigen (Fig 6A, blue bars).

The animals immunised with FD Opti-E2/HMSA showed the highest SFU (> 2,290 SFU/million cells) of all the test animals. Sheep 10 and Sheep 11 exhibited very high responses in the ELISPOT assay (Fig 6B). The density and intensity of the signal could not be directly quantified by the ELISPOT reader. In order to include these animals in the subsequent analyses each was assigned the arbitrary value of 4,000 SFU/million cells based on the upper detection level of the ELISPOT reader. These assigned values were within one standard deviation of the average values of the ELISPOT controls stimulated with ConA and were therefore considered to be realistic estimations.

In addition, one of the animals, Sheep 12, showed a higher unstimulated response than stimulated in terms of SFU number, however as shown in Fig 6B, this anomaly could have been due to background detection of the ELISPOT reader. The freeze-dried formulation included the excipients 5% trehalose and 1% glycine which are not known to be immunogenic. We have previously used 5% trehalose and 1% PEG8000 [21] with ovalbumin protein to generate freeze-dried nanovaccine and did not see increased cell-mediated responses. Once the reconstituted vaccine is injected the excipients should be rapidly desorbed from the injection site and be metabolised or cleared from the system of the animal and therefore not have any non-specific stimulatory effect on the immune system.

All the animals injected with the Opti-E2/HMSA nanoformulation showed similar low levels of cell-mediated immunity to Opti-E2 antigen with values ranging from 213 to 500 SFU/million cells. Although the responses observed were lower compared to the OptiE2-QuilA and FD Opti-E2-HMSA immunised animals, three of the four animals show significant responses (*p*<0.05) when compared to the respective unstimulated controls (Fig 6A).

It was noted that in the HMSA only injected group, Sheep 15 and 16 showed higher SFU in the stimulated samples than unstimulated (Fig 6). One possible explanation for this is the presence of low level contaminants in the recombinant antigen preparation which non-specifically stimulate cells. Every effort was made to limit this from occurring, such as the removal of LPS, however very low contaminants could stimulate low level responses in a sporadic and animal dependent manner.

The ELISPOT data in the current study was variable and the cause of this is not readily apparent (Fig 6). A factor that may have influenced these results is the use of the same antigen for immunisation of sheep and for the subsequent ELISPOT stimulation assays. Ideally the stimulation would have used a peptide representing a defined sheep major histocompatibility complex epitope of E2. However these epitopes are not well characterised in cattle and consequently even less so in sheep thus the current study was limited to the use of whole antigen. The results also demonstrate the challenges of working with an outbred large animal system with animals with low genetic homogeneity resulting in animal to animal variation which has been observed in previous immunisation studies with sheep models [26–28]. Despite these limitations the results clearly show enhanced responses to E2 in the ideal vaccine formulation, FD Opti-E2/HMSA, compared to the unstimulated cells. These results showed there was an excellent long term Th1 memory response to immunisation with FD Opti-E2/HMSA nanovaccine formulation detectable four months after immunisation.

Importantly, this is the first study to the best of our knowledge to investigate the long term immune responses in a large animal after immunisation with silica nanoparticles as all previous studies have been performed in mice [16, 17, 21, 29].

The fact that the immune response in animals receiving the E2 nanoformulation was balanced with both humoral and cellular immunity is an encouraging result. This has also been demonstrated in sheep immunised with ovalbumin covalently conjugated to polystyrene nanobeads of 50 nm in size. In that study both cellular and humoral immunity was induced most effectively when the nanobead formulation was administered through intradermal and subcutaneous routes compared to the intramuscular route [26].

The freeze-drying process was not detrimental to the nanoformulation and in fact enhanced the levels of the cell-mediated immune responses in the animals receiving the freeze-dried formulation (Fig 6). Freeze-drying of cationically modified silica nanoparticles (28 nm in size) for gene delivery in Cos-1 cells showed that addition of either 5% trehalose or 10% glycerol conserved nanoparticle integrity and subsequent biological activity through DNA-binding and enhanced transfection efficiency [30].

Here we have demonstrated for the first time that HMSA have the capacity to act as both the antigen delivery vehicle and vaccine adjuvant in a large animal. This is a significant finding since sheep are a natural target species of BVDV-1 and good model for cattle studies. In addition, unlike previous studies using an inbred laboratory mice strain [17, 21] sheep are an outbred animal and although the immune responses can be more variable, demonstration of the feasibility of silica nanovaccine technology in a large animal model is a critical research milestone.

## Conclusion

Hollow mesoporous silica nanoparticles are an attractive new adjuvant which can be precisely tailored to accommodate different antigens. HMSA nanoparticles can have the following advantages; 1) providing stability for the protein antigen through adsorption/encapsulation and therefore improved protection from degradation in the cell environment; 2) are amenable to a freeze-drying process for increased stability at ambient temperatures during storage; 3) providing a mechanism of delivery through efficient uptake due to their size by circulating dendritic cells for subsequent antigen presentation in the lymphoid organs and 4) stimulating a balanced immune response with both humoral and cell-mediated immunity. Together these advantages demonstrate that there is considerable potential for development of recombinant subunit vaccines using silica nanoparticle formulations.

## Supporting Information

S1 Fig Particle size distribution of HMSA determined by TEM imaging. (PDF)

S2 Fig Evaluation of Opti-E2/HMSA nanoformulations after freeze-drying and 14 month’s storage at ambient temperature. (PDF)

S3 Fig Full gel image of Fig 3. (PDF)
