# Supplementary figures and images for: Immunisation of Sheep with Bovine Viral Diarrhoea Virus, E2 Protein Using a Freeze-Dried Hollow Silica Mesoporous Nanoparticle Formulation
Source: PLoS One. 2015 Nov 4;10(11):e0141870. doi: 10.1371/journal.pone.0141870 (PMC4633290; doi:10.1371/journal.pone.0141870)

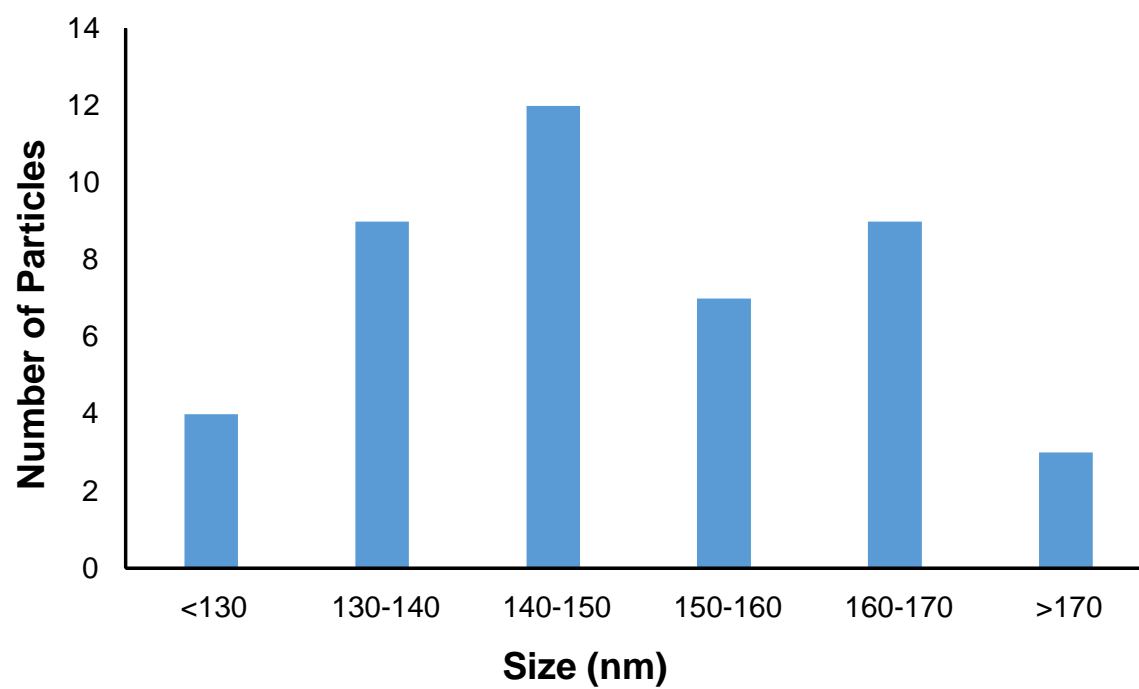

**S1 Fig.** Particle size distribution of HMSA determined by TEM imaging (total particle number = 45).

Supplement: S1 Fig — (PDF) [file pone.0141870.s001.pdf]
