# Supplementary material for: Immunisation of Sheep with Bovine Viral Diarrhoea Virus, E2 Protein Using a Freeze-Dried Hollow Silica Mesoporous Nanoparticle Formulation
Source: PLoS One. 2015 Nov 4;10(11):e0141870. doi: 10.1371/journal.pone.0141870 (PMC4633290; doi:10.1371/journal.pone.0141870)
Supplement: S2 Fig — (PDF) [file pone.0141870.s002.pdf]

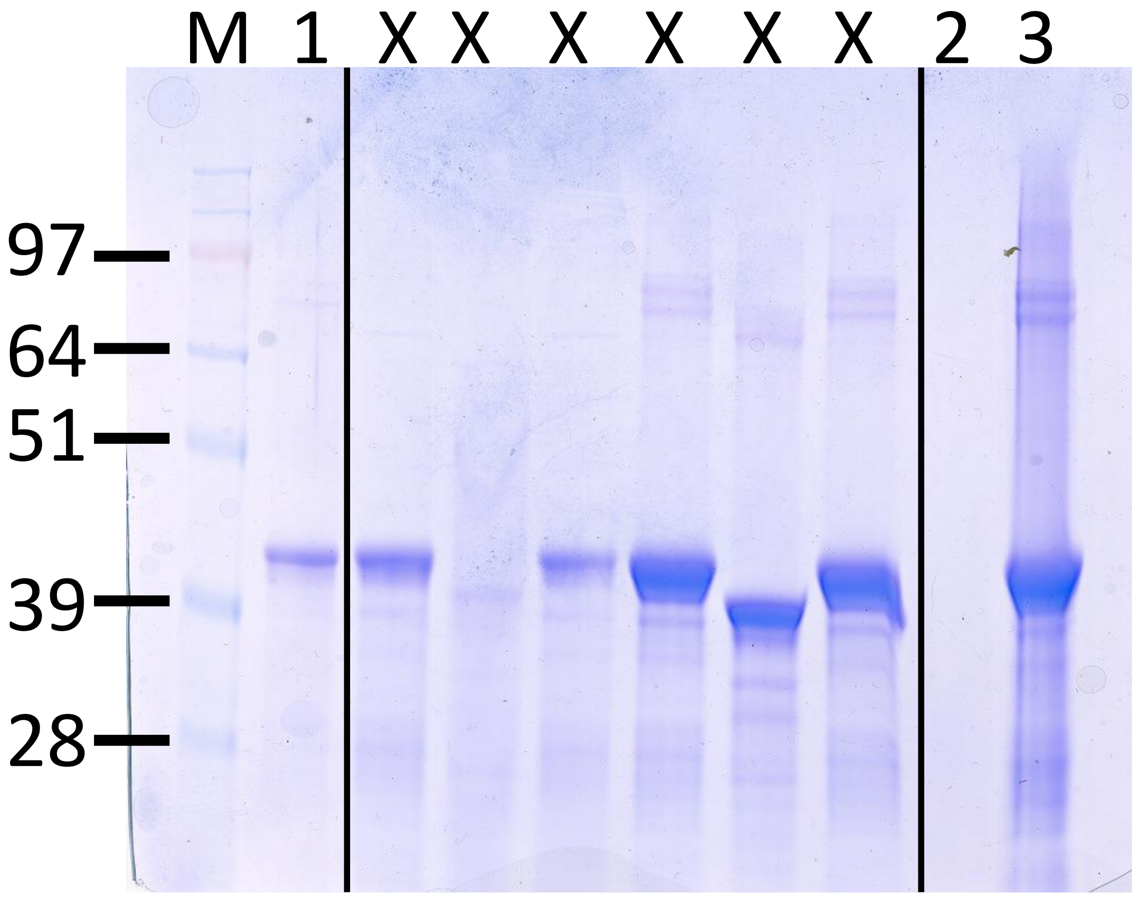

S2 Fig. Evaluation of Opti-E2/HMSA nanoformulations after freeze-drying and 14 month's storage at ambient temperature. Lane 1: Opti-E2 control (4 µg); Opti-E2/HMSA freeze-dried with Lane 2: 5% trehalose and 1% PEG8000; Lane 3: 5% trehalose and 1% glycine. Lanes X: Samples not discussed in this study.
