# Supplementary material for: Immunisation of Sheep with Bovine Viral Diarrhoea Virus, E2 Protein Using a Freeze-Dried Hollow Silica Mesoporous Nanoparticle Formulation
Source: PLoS One. 2015 Nov 4;10(11):e0141870. doi: 10.1371/journal.pone.0141870 (PMC4633290; doi:10.1371/journal.pone.0141870)
Supplement: S3 Fig — (PDF) [file pone.0141870.s003.pdf]

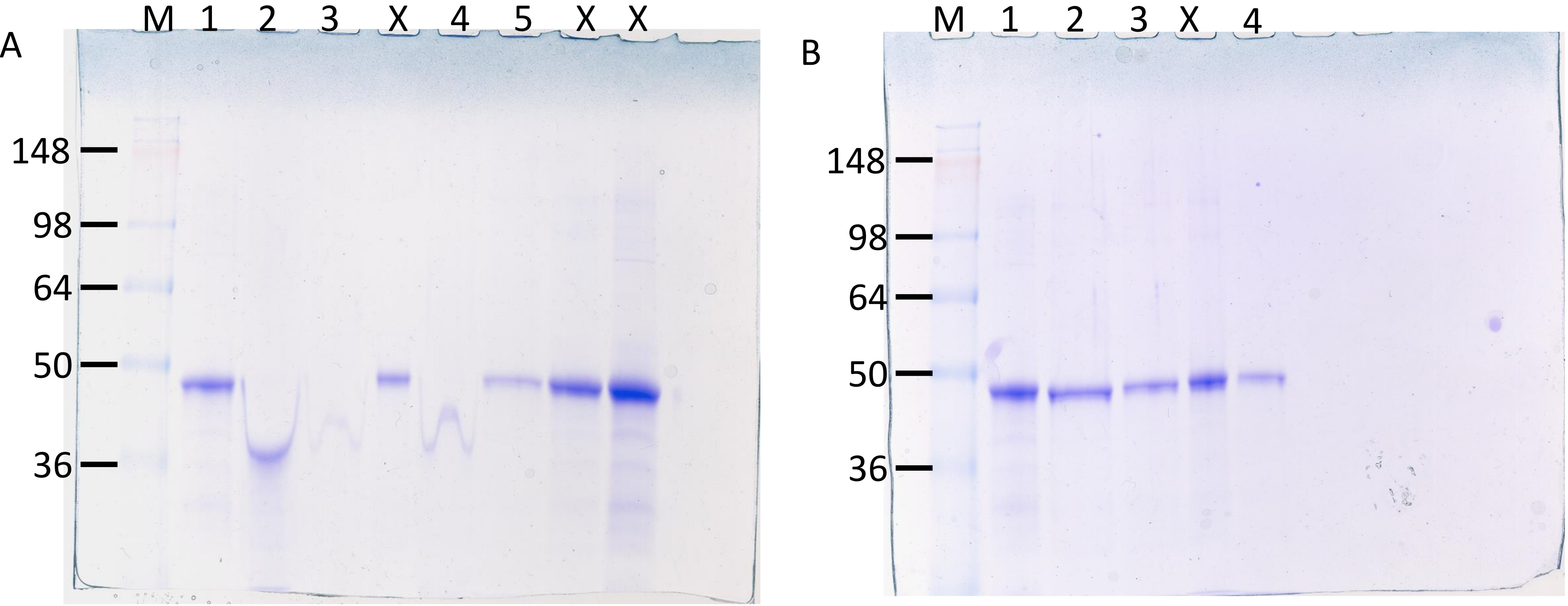

S3 Fig. Full gel images of Fig 3.

(A): Evaluation by SDS-PAGE of Opti-E2/HMSA formulations after freeze-drying with different combinations of trehalose and PEG8000. Lane 1: Opti-E2 control (4 mg); Opti-E2/HMSA freeze-dried with Lane 2: 1% PEG8000; lane 3: 5% trehalose and 0.5% PEG8000; lane 4: 5% trehalose and 0.1% PEG8000.

(B): Evaluation by SDS-PAGE of Opti-E2/HMSA formulations after freeze-drying with different combinations of trehalose and glycine. Lane 1: Opti-E2 control (4 mg); Opti-E2/HMSA freeze-dried with lane 2: 5% trehalose and 1% glycine; lane 3: 5% trehalose and 0.5% glycine; lane 4: 5% trehalose and 0.1% glycine.

Lanes X: Samples not discussed in this study.
